# Downward myocardial creep during stress PET imaging is inversely associated with mortality

**DOI:** 10.1007/s00259-024-06611-2

**Published:** 2024-01-23

**Authors:** Keiichiro Kuronuma, Robert J.H. Miller, Chih-Chun Wei, Ananya Singh, Mark H. Lemley, Serge D. Van Kriekinge, Paul B. Kavanagh, Heidi Gransar, Donghee Han, Sean W. Hayes, Louise Thomson, Damini Dey, John D. Friedman, Daniel S. Berman, Piotr J. Slomka

**Affiliations:** 1 Departments of Medicine (Division of Artificial Intelligence in Medicine), Imaging, and Biomedical Sciences, Cedars-Sinai Medical Center, 8700 Beverly Blvd, Los Angeles, CA 90048 USA; 2 Department of Cardiology, Nihon University, Tokyo, Japan; 3 Department of Cardiac Sciences, University of Calgary, Calgary, AB Canada

**Keywords:** Dynamic myocardial perfusion imaging, Myocardial flow reserve, Positron emission tomography, Regadenoson, Rubidium

## Abstract

**Purpose:**

The myocardial creep is a phenomenon in which the heart moves from its original position during stress-dynamic PET myocardial perfusion imaging (MPI) that can confound myocardial blood flow measurements. Therefore, myocardial motion correction is important to obtain reliable myocardial flow quantification. However, the clinical importance of the magnitude of myocardial creep has not been explored. We aimed to explore the prognostic value of myocardial creep quantified by an automated motion correction algorithm beyond traditional PET-MPI imaging variables.

**Methods:**

Consecutive patients undergoing regadenoson rest-stress [^82^Rb]Cl PET-MPI were included. A newly developed 3D motion correction algorithm quantified myocardial creep, the maximum motion at stress during the first pass (60 s), in each direction. All-cause mortality (ACM) served as the primary endpoint.

**Results:**

A total of 4,276 patients (median age 71 years; 60% male) were analyzed, and 1,007 ACM events were documented during a 5-year median follow-up. Processing time for automatic motion correction was < 12 s per patient. Myocardial creep in the superior to inferior (downward) direction was greater than the other directions (median, 4.2 mm vs. 1.3–1.7 mm). Annual mortality rates adjusted for age and sex were reduced with a larger downward creep, with a 4.2-fold ratio between the first (0 mm motion) and 10th decile (11 mm motion) (mortality, 7.9% vs. 1.9%/year). Downward creep was associated with lower ACM after full adjustment for clinical and imaging parameters (adjusted hazard ratio, 0.93; 95%CI, 0.91–0.95; *p* < 0.001). Adding downward creep to the standard PET-MPI imaging model significantly improved ACM prediction (area under the receiver operating characteristics curve, 0.790 vs. 0.775; *p* < 0.001), but other directions did not (*p* > 0.5).

**Conclusions:**

Downward myocardial creep during regadenoson stress carries additional information for the prediction of ACM beyond conventional flow and perfusion PET-MPI. This novel imaging biomarker is quantified automatically and rapidly from stress dynamic PET-MPI.

**Supplementary Information:**

The online version contains supplementary material available at 10.1007/s00259-024-06611-2.

## Introduction

Positron emission tomography (PET) myocardial perfusion imaging (MPI) provides an assessment of absolute myocardial blood flow (MBF) and myocardial blood reserve (MFR) along with traditional PET-MPI variables like myocardial ischemia, left ventricular ejection fraction (LVEF), and left ventricular end-diastolic volume (LVEDV). Impaired MFR is indicative of coronary vasculature impairment and exhibits a strong association with major adverse cardiac events and mortality [1]. Since cardiac motion often occurs on stress-dynamic PET-MPI and can potentially confound MBF measurements, motion correction is important to obtain reliable myocardial flow quantification on PET-MPI [2]. This cardiac motion during vasodilator stress dynamic MPI is primarily related to myocardial creep [3].

Myocardial creep was originally described as an upward creep and a source of artifact on exercise stress single photon emission computed tomography (SPECT) MPI using thallium-201 [4]. The mechanism of upward creep was thought to be caused by a gradual decrease in lung volumes after the termination of exercise stress with a diminishing depth of respiration [4]. On the other hand, in vasodilator stress MPI, the heart initially moves downward (inferiorly) after the administration of vasodilator and then moves upward (superiorly) after the termination of vasodilator [5–7]. In recent studies, using rubidium-82 ([^82^Rb]Cl) dynamic PET-MPI with regadenoson, myocardial creep defined visually as misregistration of at least one third of the left ventricular wall width occurred in half of the patients, primarily in the downward (inferior) direction [8, 9]. We developed an automated 3D motion correction algorithm, which can quantify the magnitude of myocardial creep [10]. However, the clinical importance of these measures has not been studied.

The aim of the present study is to assess the association between the magnitude of myocardial creep determined by an automated motion correction algorithm and all-cause mortality (ACM), and to explore the incremental prognostic value of myocardial creep beyond conventional PET-MPI variables including MFR.

## Materials and methods

### Study population

A total of 4,298 consecutive patients from Cedars-Sinai Medical Center, who underwent regadenoson rest-stress [^82^Rb]Cl PET-MPI between January 2010 and December 2018, and provided informed consent, were enrolled in the study. The study was approved by Cedars Sinai Medical Center’s institutional review board. This study adheres to the ethical standards of the Declaration of Helsinki.

### Clinical data

Demographic and clinical data such as age, sex, body mass index (BMI), history of previous myocardial infarction (MI), family history of CAD, percutaneous coronary intervention (PCI), coronary artery bypass graft surgery (CABG), diabetes, dyslipidemia, and hypertension were recorded according to the status at the time the scan. Revascularization information was extracted from hospital records adjudicated by experienced cardiologists.

### Imaging acquisition

Rest and pharmacological stress PET-MPI studies utilizing [^82^Rb]Cl were performed with a Biograph 64 PET/CT scanner (Siemens Healthcare, Erlangen, Germany) or GE Discovery 710 (GE Healthcare, Waukesha, Wisconsin) scanners. Directly prior to the injection of weight-based dose of 925–1,850 MBq (25–50 mCi) of [^82^Rb]Cl, a 6-min rest list-mode acquisition was initiated. Regadenoson was utilized to induce pharmacological stress, and a 6-min stress list-mode acquisition was initiated at the same time along with the [^82^Rb]Cl infusion of the same dose as for the rest scan. As previously detailed, before each rest and stress PET scanning, a low-dose CT scan was obtained for attenuation correction [11].

### Automated myocardial contour positioning

Myocardial contours were positioned automatically from the reconstruction of image data from the last 4 min of the 6-min list-mode acquisition by QPET software (Cedars-Sinai, Los Angeles, CA) [12]. Transaxial PET image reorientation into the short axis was automatically performed prior to motion correction.

### Automated motion correction

The motion correction algorithm has been recently described [10]. In brief, the algorithm for constructing the 3D geometric model of the left and right ventricles is based on the summed image of the list mode acquisition (without the first 2 min). The method aligns individual image frames to this model using 3D rigid-body translations in three directions: lateral-septal, basal-apical, and superior-inferior. Three phases (blood-pool phase, myocardium uptake phase, and the transition between two) were automatically identified based on time-activity curves. Three keyframes in each of the 3 phases were independently aligned to the model using simplex maximization of similarity function. Linear blending was used for the frames between the keyframes. All corrections were performed fully automatically in batch mode.

The magnitude of myocardial creep was defined as the maximum myocardial motion in each direction (in mm) at stress within 60 s after tracer injection (first pass), since maximum myocardial motion by pharmacological stress MPI occurred within 60 s after tracer injection in previous studies [6, 9, 13].

### Conventional MPI variable quantification

QPET software (Cedars-Sinai, Los-Angeles, CA) was used to automatically derive myocardial perfusion and function quantitative variables consisting of rest and stress total perfusion deficit (TPD), LVEF, and end-diastolic end-systolic left ventricular volumes [12].

### MBF quantification

List-mode data contained 6-min of the count acquisition and were reconstructed into 16 frames (12 × 10, 2 × 30, 1 × 60 and 1 × 120 s). Subsequently, clinical QPET software (Cedars Sinai, Los Angeles, CA) was applied to calculate rest and stress MBF with a 1-tissue compartment kinetic model [14]. MBF and spillover fraction from blood to myocardium were included in the model computations. Stress and rest MBF values were computed in mL/g/min for individual polar-map samples. MFR was calculated by taking the ratio of stress MBF over rest MBF. All stress and rest MBF quantification utilized automated motion correction.

### Study end point

Mortality status was determined using internal hospital records, the Social Security Death Index, National Death Index, and the California Non-Comprehensive Death File [15].

### Statistical analysis

Categorical variables are presented as numbers and percentages and were compared by the χ^2^ test. Continuous variables are presented as mean ± standard deviation (SD) or median values (IQR) and were compared by the Student T test or Mann-Whitney U test. The Kolmogorov-Smirnov test was used to verify a normal distribution. Spearman correlation coefficients were used for verifying associations among the continuous variables. Annualized mortality rates adjusted by age and biological sex were computed across decile of downward creep. Kaplan-Meier survival curves after the full adjustment, stratified by median value of downward creep (4.2 mm), were used to assess the primary outcome of ACM and compared using the log-rank test. Since previous study has shown that MFR is a stronger predictor of cardiovascular mortality than MBF, we used MFR rather than MBF for the prognostic analysis [16].

Associations between downward creep and ACM were assessed using a Cox regression. All multivariable models consisted of the following variables: age, sex, body mass index, hypertension, hyperlipidemia, diabetes, smoking, prior history of CAD (prior history of MI, PCI, or CABG), cerebral stroke, peripheral vascular disease, anginal chest pain, shortness of breath, early revascularization (< 90 days after the PET study), stress TPD, rest TPD, stress-rest change in LVEF, rest LVEF, rest LVEDV, heart rate (HR) response (peak–rest HR, bpm) [17], systolic blood pressure (BP) response (peak-rest systolic BP, mmHg), diastolic BP response (peak-rest diastolic BP, mmHg) [18], and MFR. We checked multicollinearity for the variables for the adjustment by variance inflation factors (VIF) and these were not significant multicollinearity (all VIF < 4) [19]. We evaluated the proportional hazards assumption by the Schoenfeld residuals test, and the variable of age violated the assumption. However, the proportional assumption of those variables was not visually violated on the Schoenfeld residual plot, and when we modeled these variables as time-varying covariates, the results for the myocardial creep were similar. To assess whether the association between downward creep and ACM changed over time in relation to improvement in medical treatment, the analysis was repeated for patients who underwent PET-MPI from 2014 to 2018. Global χ2 analyses and likelihood ratios test were used to assess the incremental fit of the model that incorporates downward creep, as opposed to the model with conventional MPI variables (stress TPD, rest TPD, stress-rest change in LVEF, rest LVEF, rest LVEDV, HR response, systolic BP response, and diastolic BP response) and either MFR or stress MBF alone. Receiver-operating characteristic analysis based on Cox-derived models and pairwise comparisons according to DeLong et al. to compare areas under the curves (AUC) were performed [20]. A two-sided *p*-value of < 0.05 was considered significant. R version 4.2.0 (R Foundation for Statistical Computing, Vienna, Austria) or STATA version 16 (StataCorp LP, College Station, TX) was used to perform statistical analyses.

## Results

### Patient characteristics and outcome

A total of 4,276 patients (median age 71 years; 60% male) were analyzed, due to the exclusion of patients with missing essential data (e.g., stress and rest heart rate or blood pressure), and 1,007 ACM events were documented during a 4.9 year [IQR, 2.9–6.7 years] median follow-up. Table 1 shows the baseline characteristics of the patient population. Patients who died were older (median age, 77 vs. 70 years, *p* < 0.001) and exhibited a higher prevalence of hypertension, diabetes, and a history of CAD (all *p* < 0.001) (Table 1).

**Table 1:** Baseline characteristics

Number	Overall	ACM	No ACM	*p* value
	4,276	1,007	3,269	
Age, y	71 [64, 79]	77 [68, 84]	70 [63, 77]	< 0.001
Male, %	59.6	61.8	58.9	0.106
Body mass index, kg/m2	27.4 [24.1, 31.6]	26.0 [23.0, 30.3]	27.8 [24.5, 32.0]	< 0.001
Hypertension, %	78.3	82.5	76.9	< 0.001
Hyperlipidemia, %	68.3	63.8	69.7	< 0.001
Diabetes, %	34.6	41.8	32.4	< 0.001
Smoking, %	7.6	7.0	7.8	0.378
History of CAD, %	35.6	45.8	32.5	< 0.001
Cerebral stroke, %	10.0	12.2	9.3	0.008
PVD, %	9.1	11.9	8.2	0.001
Anginal chest pain, %	53.5	48.2	55.1	< 0.001
Shortness of breath, %	47.8	49.4	47.4	0.279
Early revascularization, %	9.2	11.5	8.4	0.004
Stress TPD, %	3.4 [1.3, 9.1]	6.3 [2.2, 15.4]	2.9 [1.1, 7.4]	< 0.001
Rest TPD, %	0.4 [0.0, 2.1]	1.2 [0.1, 6.0]	0.2 [0.0, 1.6]	< 0.001
LVEF at stress, %	68.3 [56.9, 76.0]	60.1 [43.9, 70.2]	70.1 [60.8, 77.1]	< 0.001
LVEF at rest, %	64.8 [53.8, 72.3]	57.0 [41.9, 67.7]	66.4 [57.6, 73.2]	< 0.001
Stress-rest change in LVEF, %	3.4 [0.2, 6.2]	2.5 [-0.7, 5.2]	3.7 [0.5, 6.4]	< 0.001
LVEDV at stress, mL	96 [74, 125]	103 [75, 139]	94 [73, 121]	< 0.001
LVEDV at rest, mL	86 [66, 113]	94 [67, 128]	84 [65, 108]	< 0.001
LVESV at stress, mL	30 [19, 51]	39 [24, 72]	28 [18, 46]	< 0.001
LVESV at rest, mL	30 [19, 49]	39 [23, 69]	29 [18, 44]	< 0.001
MBF at stress, mL/g/min	2.44 [1.81, 3.11]	2.03 [1.45, 2.76]	2.55 [1.95, 3.18]	< 0.001
MBF at rest, mL/g/min	1.09 [0.85, 1.37]	1.14 [0.89, 1.44]	1.08 [0.84, 1.34]	< 0.001
MFR	2.20 [1.70, 2.79]	1.75 [1.36, 2.26]	2.34 [1.84, 2.92]	< 0.001
Rest HR, bpm	68 [60, 77]	71 [63, 81]	67 [60, 76]	< 0.001
Peak HR, bpm	89 [79, 100]	85 [75, 96]	90 [80, 102]	< 0.001
HR response, bmp	20 [11, 29]	12 [5, 20]	22 [13, 30]	< 0.001
Rest systolic BP, mmHg	135 [122, 149]	134 [120, 150]	135 [123, 149]	0.118
Peak systolic BP, mmHg	115 [102, 129]	114 [98, 129]	116 [103, 130]	< 0.001
Systolic BP response, mmHg	-20 [-32, -8]	-21 [-34, -9]	-19 [-31, -7]	0.011
Rest diastolic BP, mmHg	72 [64, 81]	69 [61, 79]	73 [65, 82]	< 0.001
Peak diastolic BP, mmHg	54 [46, 63]	50 [41, 59]	55 [47, 64]	< 0.001
Diastolic BP response, mmHg	-18 [-25, -10]	-20 [-27, -11]	-17 [-25, -10]	< 0.001
**Stress maximum myocardial motion during first pass**				
Superior to inferior, mm	4.2 [2.2, 6.7]	2.9 [1.1, 4.8]	4.7 [2.6, 7.1]	< 0.001
Lateral to septal, mm	1.7 [0.3, 3.2]	1.3 [0.0, 2.8]	1.8 [0.5, 3.3]	< 0.001
Basal to apical, mm	1.3 [0.0, 3.0]	1.1 [0.0, 2.8]	1.4 [0.0, 3.0]	0.020
**Rest maximum myocardial motion during first pass**				
Superior to inferior, mm	0.8 [0.0-2.3]	0.6 [0.0–2.0]	0.9 [0.0-2.4]	< 0.001
Lateral to septal, mm	0.6 [0.0-1.7]	0.5 [0.0-1.6]	0.7 [0.0-1.7]	0.010
Base to apex, mm	0.9 [0.0-2.1]	0.8 [0.0-1.6]	0.9 [0.0-2.1]	0.693

Values are shown as median [25th, 75th percentiles] or number (%) of patients. ACM, all-cause mortality; BP, blood pressure; CAD, coronary artery disease; HR, heart rate; LVEDV, left ventricular end-diastolic volume; LVEF, left ventricular ejection fraction; LVESV, left ventricular end-systolic volume; MBF, myocardial blood flow; MFR, myocardial flow reserve; PVD, peripheral vascular disease; TPD, total perfusion deficit

### PET MPI findings and outcomes

**Fig. 1:**
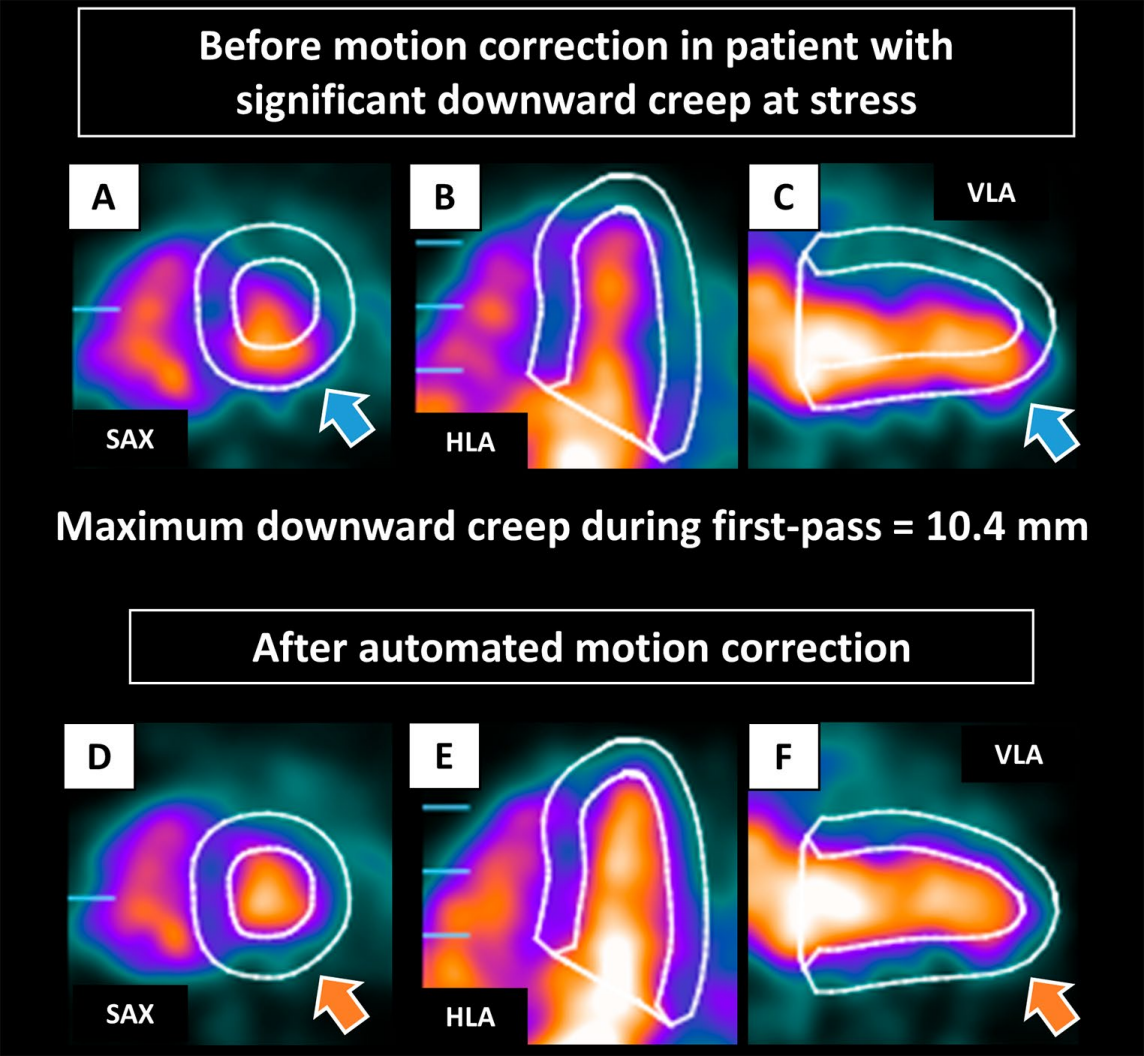
Case example of early dynamic images before (**A-C**) and after (**D-F**) automated motion correction in patient with significant downward creep at stress. The LV contours before motion correction were automatically positioned from the static imaging. Before correction, the inferior LV contour overlaps substantially with the activity of the LV blood pool, and the anterior LV contour is far from the actual LV myocardium (blue arrows). Those were corrected after automated motion correction (orange arrows). HLA, horizontal long axis; LV, left ventricle; SAX, short axis; VLA, vertical long axis

Case example of early dynamic images before and after automated motion correction for a patient with significant downward creep is shown in Fig. 1. All automated motion corrections were processed within 12 s per case for each stress and rest dynamic scan. All continuous variables were not normally distributed. Myocardial creep at stress in the superior to inferior direction (downward creep) was greater than the other directions (median 4.2 mm for downward creep, 1.7 mm for lateral to septal direction, and 1.3 mm for basal to apical direction). The myocardial motion at rest in all directions was minimal (median < 1 mm for all directions). Patients with ACM had lower downward creep compared to those without ACM (median 2.9 vs. 4.7 mm) (Table 1). Patients with ACM had significantly higher TPD, LVEDV, and left ventricular end-systolic volume and lower LVEF, stress MBF, and MFR (Table 1).

**Fig. 2:**
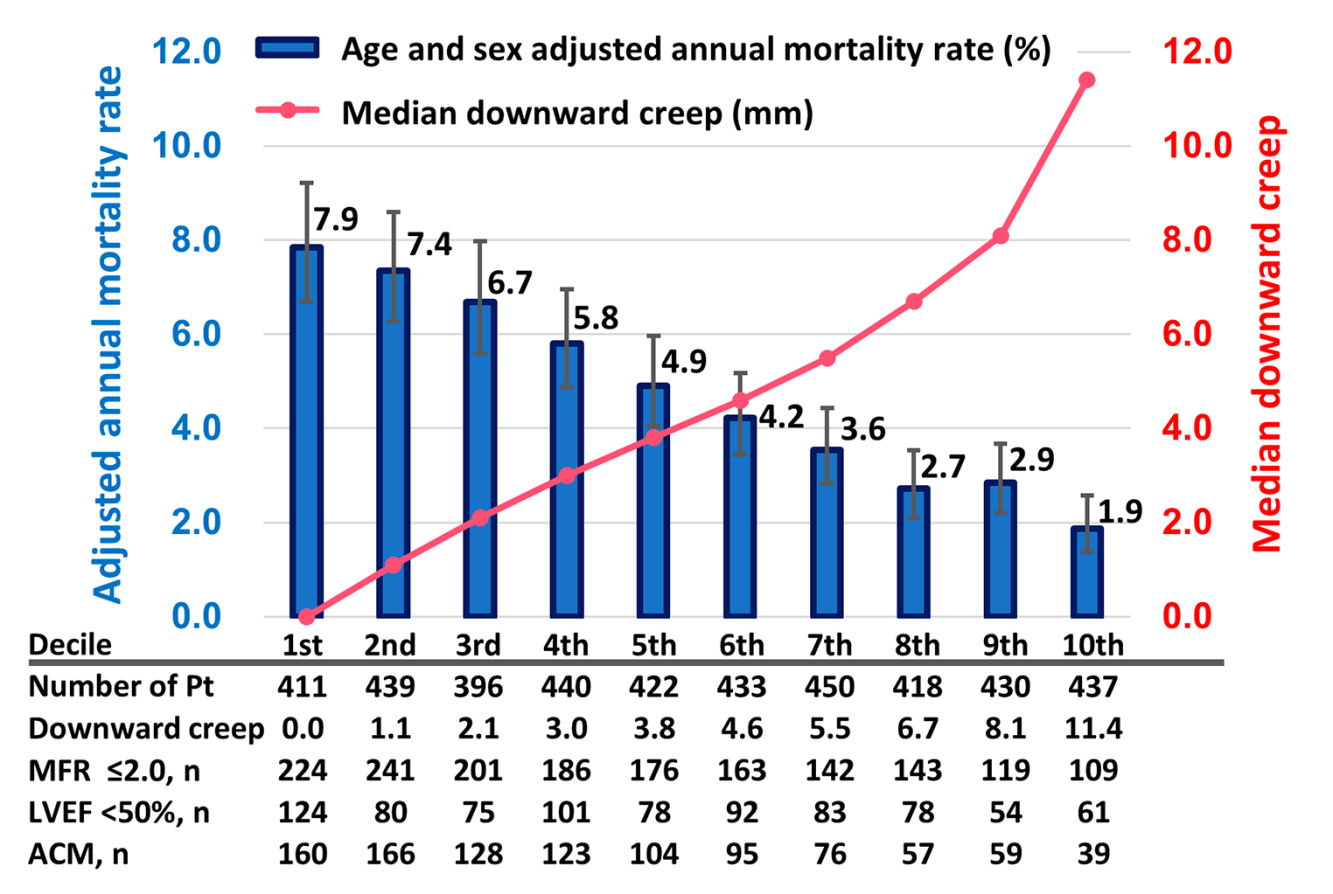
Annualized mortality rate adjusted by age and sex and deciles of downward creep. The left y axis and blue bars indicate the adjusted annual mortality rates (%). The right y axis and pink line indicate median downward creep (mm)

Figure 2 shows annual mortality rates adjusted for age and sex, distributed across the deciles of downward creep. As downward creep increased, there was a corresponding reduction in annual mortality rates. Annual mortality rates were 4.2-fold higher for patients in the lowest decile (1st) compared to those in the highest decile (10th), with corresponding rates of 7.9% vs. 1.9%, respectively (*p* < 0.001).

### Relationships between downward creep and other MPI variables

Supplemental Fig. 1 shows correlation coefficients between all continuous variables. There were weak correlations (*r*= -0.19 to 0.24) between downward creep and age, BMI, and other PET-MPI variables including MFR. The patients with higher downward creep tended to have higher MFR. Downward creep was also weakly correlated with lateral-septal and basal-apical directions of myocardial motion (*r* = 0.26 and *r* = 0.22, respectively) (Supplemental Fig. 1).

### Kaplan-Meier analysis

**Fig. 3:**
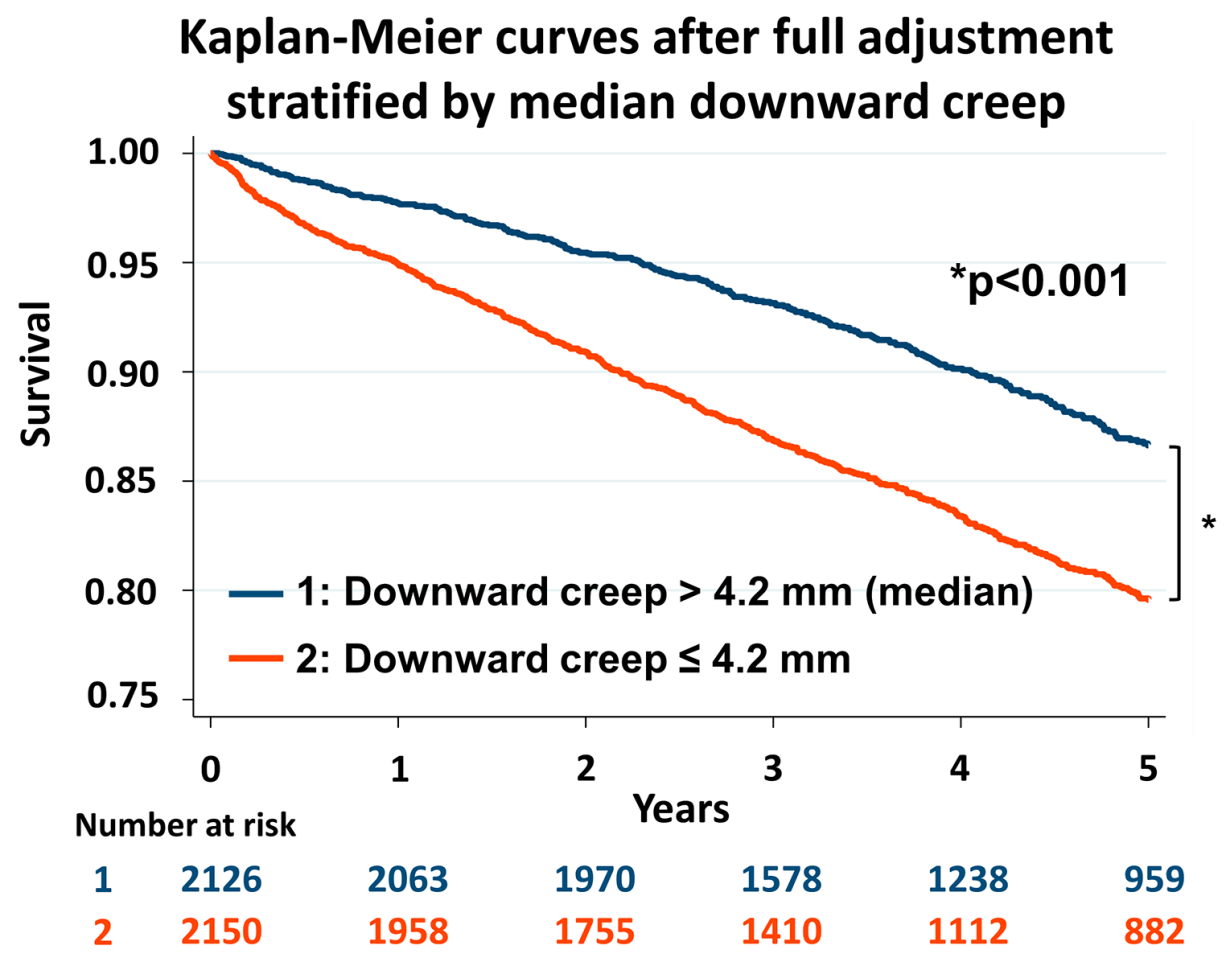
Kaplan-Meier curves stratified by median downward creep adjusted by following variables: age, sex, body mass index, hypertension, hyperlipidemia, diabetes, smoking, prior history of coronary artery disease, cerebral stroke, peripheral vascular disease, anginal chest pain, shortness of breath, early revascularization, stress TPD, rest TPD, stress-rest change in LVEF, rest LVEF, rest LVEDV, heart rate response, systolic BP response, diastolic BP response, and myocardial flow reserve. BP, blood pressure; LVEDV, left ventricular end-diastolic volume; LVEF, left ventricular ejection fraction; TPD, total perfusion deficit

Kaplan-Meier survival curves after the full adjustment for ACM were drawn according to the median value of downward creep (4.2 mm) (Fig. 3). Patients with low downward creep (≤ 4.2 mm) had higher mortality risk compared to those with high downward creep (> 4.2 mm) (*p* < 0.001) (Fig. 3).

### Cox proportional hazards analysis

**Fig. 4:**
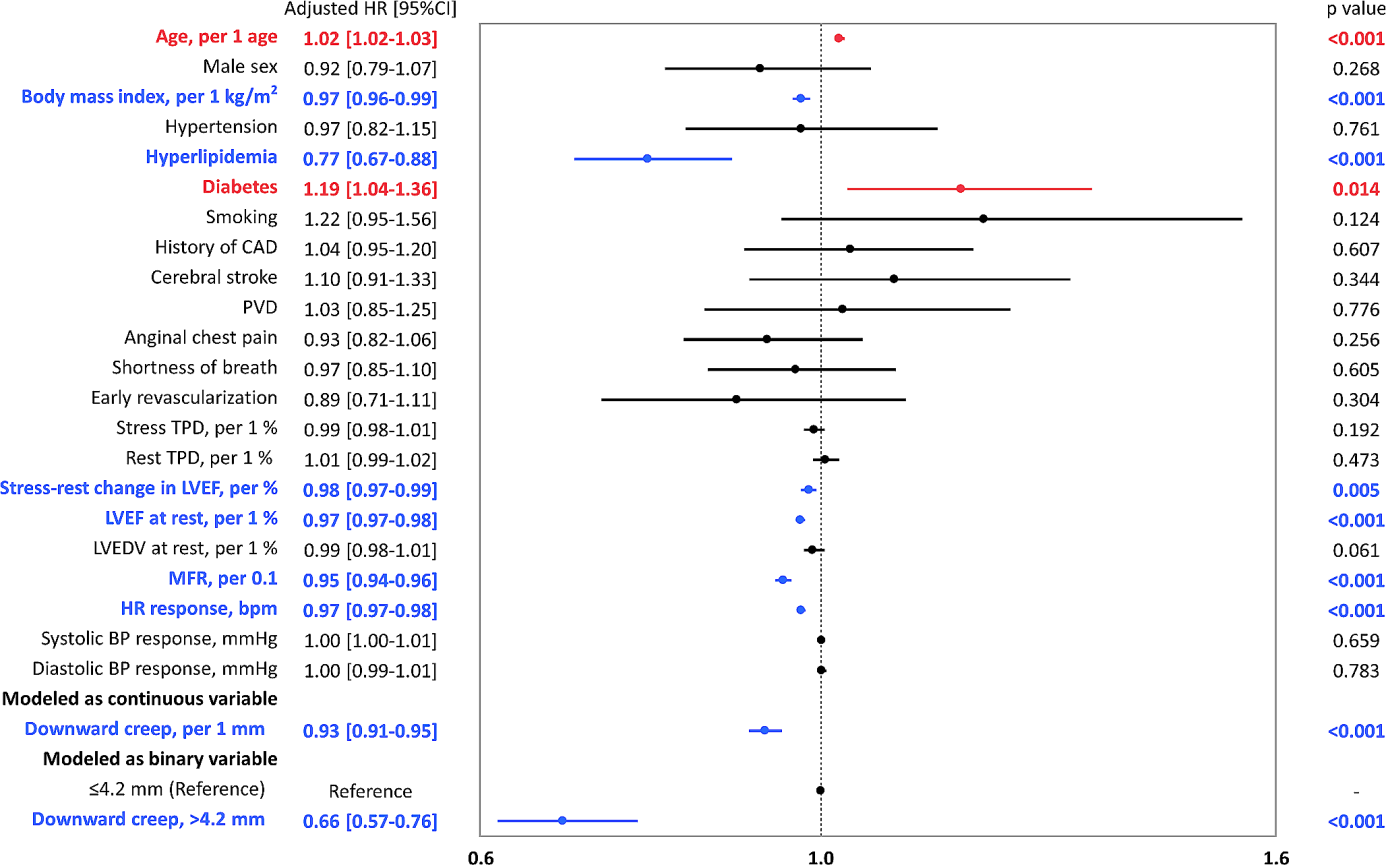
Forest plot of multivariable adjusted HRs for ACM. Blue bars indicate the variables are significantly and negatively associated with ACM. Red bars indicate the variables are significantly and positively associated with ACM. Black bars indicate the variables are not significantly associated with ACM. ACM, all-cause mortality; BMI, body mass index; BP, blood pressure; CAD, coronary artery disease; HR, hazard ratio; LVEDV, left ventricular end-diastolic volume; LVEF, left ventricular ejection fraction; MFR, myocardial flow reserve; PVD, peripheral vascular disease; TPD, total perfusion deficit

Figure 4 shows the adjusted HRs for prediction of ACM from the Cox regression analysis. Downward creep demonstrated an independent association with ACM even after multivariable adjustment (adjusted hazard ratio 0.93 /1 mm; 95%CI, 0.91–0.95; *p* < 0.001) (Fig. 4). The adjusted hazard ratio of downward creep over the median value (4.2 mm) compared to low downward creep (≤ 4.2 mm) was 0.66 (95%CI, 0.57–0.76; *p* < 0.001) (Fig. 4). The results of unadjusted and adjusted HRs for ACM in each direction of maximum myocardial motion during first pass are shown in Supplemental Table 1. When downward myocardial creep was modeled with restricted cubic splines, the association between downward myocardial creep and ACM was almost linear (Supplemental Fig. 2).

When using normalized downward creep (downward creep divided by body surface area), similar results were obtained (adjusted hazard ratio 0.87 per 1 mm/m^2^; 95%CI, 0.83–0.90; *p* < 0.001). The adjusted hazard ratio of normalized downward creep over the median (> 2.23 mm/m^2^) compared to low downward creep (≤ 2.23 mm/m^2^) was 0.72 (95%CI, 0.63–0.83; *p* < 0.001).

When limited to the patients who underwent the PET study from 2014 to 2018, similar results were obtained (adjusted hazard ratio 0.91 per 1 mm; 95%CI, 0.88–0.94; *p* < 0.001). The adjusted hazard ratio of downward creep over the median (> 4.5 mm) compared to low downward creep (≤ 4.5 mm) was 0.62 (95%CI, 0.50–75; *p* < 0.001).

### Incremental value of downward creep over conventional MPI variables and MFR

**Fig. 5:**
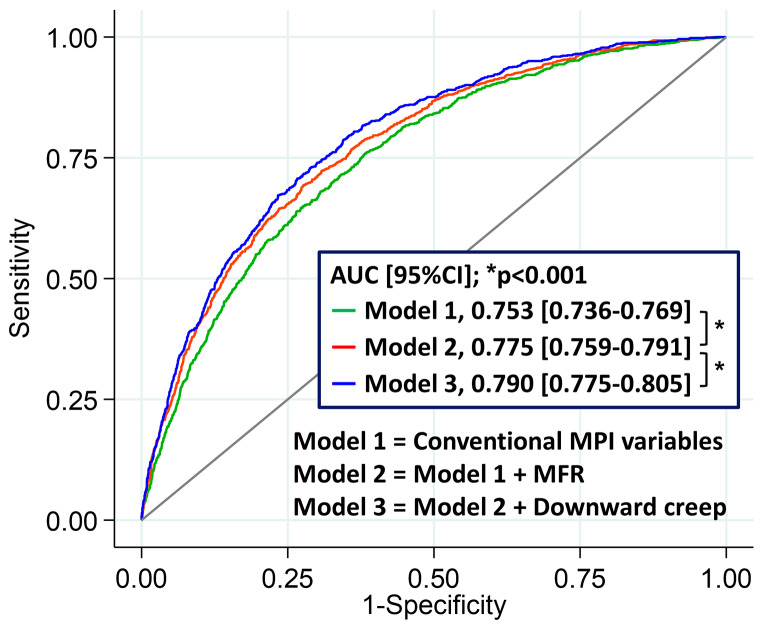
Incremental value of downward creep to predict mortality beyond conventional MPI variables and MFR. MFR, myocardial flow reserve; MPI, myocardial perfusion image

The global χ^2^ for the model adding downward creep to conventional MPI variables (stress TPD, rest TPD, stress-rest change in LVEF, rest LVEF, rest LVEDV, HR response, systolic BP response, and diastolic BP response) and MFR was significantly higher than that for conventional MPI variables and MFR alone (*p* < 0.001) (Supplemental Fig. 3). When using stress MBF instead of MFR, similar results were observed, with significantly higher global χ^2^ for the model with downward creep (*p* < 0.001) (Supplemental Fig. 4). The addition of downward creep significantly enhanced the predictive accuracy of the model for ACM when compared to the model with traditional MPI variables and MFR alone (AUC [95%CI], 0.79 [0.78–0.81] vs. 0.78 [0.76–0.79]; *p* < 0.001) (Fig. 5). Adding other direction of myocardial motion did not improve the predictive performance (AUC [95%CI], 0.78 [0.76–0.79] vs. 0.78 [0.76–0.79]; *p* = 0.55 for lateral to septal direction and AUC [95%CI], 0.78 [0.76–0.79] vs. 0.78 [0.76–0.79]; *p* = 0.88 for basal to apical direction).

## Discussion

We sought to evaluate the association between downward creep measured by an automated algorithm and ACM. The major findings from this study were as follows: (1) the magnitude of myocardial creep in the downward direction exceeded that in the other directions and there was a gradual reduction in the annualized mortality rate as downward creep increased, (2) downward creep parameter demonstrated an independent association with ACM after adjustment for all MPI imaging variables including MFR, (3) the model including downward creep significantly improved prediction of ACM. Downward creep can be quantified automatically using dedicated motion correction software and is a novel biomarker with potential clinical utility.

To our knowledge this is the first study to evaluate the association between myocardial creep during stress dynamic MPI and clinical outcomes. There was a gradual decrease in annualized mortality rate with increasing downward creep, with a 4.2-fold increase in ACM risk across deciles of downward creep (Fig. 2). Additionally, we went on to demonstrated that downward creep was independently associated with ACM after adjusting for the important PET-MPI imaging and clinical variables. We also identified that downward creep significantly improved risk stratification for ACM beyond these traditional PET-MPI variables. Our findings suggest that downward creep is a potentially valuable, novel feature to consider when estimating a patient’s risk of ACM.

The study is the largest to date to characterize downward-creep in vasodilator stress dynamic PET-MPI [6–9, 13]. We utilized an automated algorithm to perform 3D motion correction for all images by batch processing, which allowed us to evaluate the myocardial creep objectively and quantitatively. Using this algorithm, the magnitude of downward creep can be obtained fully automatically and rapidly (< 12 s) without extra radiation or image acquisition time. Previous studies of dynamic PET-MPI with regadenoson showed that myocardial creep defining visually as decreasing misalignment of over one third of the LV wall width was observed in 52% (54/104) of patients [8] and in 48% (31/64) of patients [9]. In line with those studies, median value of downward creep was 4.2 mm. Our finding that the cardiac motion during stress dynamic MPI occurs primarily in the inferior direction is consistent with previous studies [7–9]. Downward creep was only weakly correlated with age, BMI, or other MPI variables (Supplemental Fig. 1). These findings suggest that downward creep is a common finding on stress PET-MPI which can be quantified rapidly in a fully automated fashion and has weak or no correlation with other MPI variables.

Although the present observational study cannot reveal the underlying mechanism of downward creep on vasodilator stress dynamic MPI, one potential mechanism has been previously hypothesized. Myocardial creep may be caused by temporary increase in respiration by vasodilator, and patients with normal pulmonary function may have greater changes in lung volume than those with impaired pulmonary function. In healthy volunteers, adenosine has been shown to induce an increase in respiratory rate, tidal volume, and the partial pressure of oxygen [21, 22]. In contrast, in patients with moderate or severe chronic obstructive pulmonary disease, no differences in respiratory rate, forced vital capacity, and oxygen saturation were observed between patients receiving regadenoson and placebo [23]. Our findings might suggest that the magnitude of downward creep represents responsiveness to regadenoson and that patients with normal pulmonary function have greater downward creep than those with impaired pulmonary function.

Our study has several limitations. Since it is an observational study, the mechanism of our findings could not be established. However, our results are based on a large cohort and demonstrate that the downward myocardial creep is a robust biomarker of mortality. Further studies are warranted to clarify the detailed mechanism of downward creep. It is possible that patient’s body motion is included in the quantification of myocardial creep in some cases; however, most of the myocardial creep is likely due to variability in respiratory excursion by pharmacological vasodilator [3]. In fact, median myocardial motion on rest imaging was less than 1 mm (Table 1). Additionally, myocardial creep measurements were limited only to first pass (the first 60 s after tracer injection), which would minimize contamination from patient motion. We also explored associations between septal-lateral and basal-apical motion with ACM and found that the former was weakly associated with ACM and the latter was not (Supplemental Table 1), and these motions did not have incremental value for predicting ACM in ROC analysis. Further, in the present study, regadenoson was used for pharmacological stress, and it is unknown whether downward creep can be utilized to predict ACM when other stress agents such as adenosine or dobutamine are used. However, regadenoson is the most used pharmacological stress agent for MPI, accounting for approximately 84% of the scans in 2013 [24]. Since our study was conducted as a single center retrospective observational study, our findings may need further validation in other cohorts. Detailed medical management changes or use of device therapies such as implantable cardioverter-defibrillator and cardiac resynchronization therapy after the PET study were unknown. We were not able to determine cardiovascular mortality due to the large, retrospective nature of the study; however, the exact identification of cause of death has significant limitations [25]. Lastly, we lacked information regarding the presence of obstructive or restrictive lung disease or frailty which may have elucidated the mechanism.

## Conclusion

The downward myocardial creep is independently associated with ACM and improved risk stratification over standard clinical and PET-MPI variables including myocardial blood flow measurements. Downward creep can be obtained fully automatically and rapidly from stress dynamic PET-MPI and represents a new imaging biomarker to improve mortality risk prediction.

### Electronic supplementary material

Below is the link to the electronic supplementary material.

Supplementary Material 1

## Data Availability

All data generated or analysed during this study are included in this published article and the supplementary information files.
