# Supplementary material for: Downward myocardial creep during stress PET imaging is inversely associated with mortality
Source: Eur J Nucl Med Mol Imaging. 2024 Jan 23;51(6):1622–31. doi: 10.1007/s00259-024-06611-2 (PMC11042981; doi:10.1007/s00259-024-06611-2)
Supplement: Supplementary file 1 — Supplementary Material 1 [file 259_2024_6611_MOESM1_ESM.docx]

**Downward myocardial creep during stress PET imaging is inversely associated with mortality**

Keiichiro Kuronuma, MD,^a,b^ Robert J.H. Miller, MD,^a,c^ Chih-Chun Wei, MS,^a^ Ananya Singh, MS,^a^ Mark H. Lemley, BS,^a^ Serge D. Van Kriekinge, PhD,^a^ Paul B. Kavanagh, MS,^a^ Heidi Gransar, MS,^a^ Donghee Han, MD,^a^ Sean W. Hayes, MD,^a^ Louise Thomson, MD,^a^ Damini Dey, PhD,^a^ John D. Friedman, MD,^a^ Daniel S. Berman, MD,^a^ and Piotr J. Slomka, PhD,^a^

1. Departments of Medicine (Division of Artificial Intelligence in Medicine), Imaging, and Biomedical Sciences, Cedars-Sinai Medical Center, Los Angeles, California, United States
2. Department of Cardiology, Nihon University, Tokyo, Japan
3. Department of Cardiac Sciences, University of Calgary, Calgary, Alberta, Canada

**Corresponding author:**

Piotr J. Slomka, PhD

Department of Medicine, Cedars-Sinai Medical Center

8700 Beverly Blvd. Los Angeles, CA 90048

Email: Piotr.Slomka@cshs.org

Tel: +1 310 423 4348, Fax: +1 310 423 0173

ORCID iD: <https://orcid.org/0000-0002-6110-938X>

**SUPPLEMENTAL MATERIAL**

Supplemental Table 1 Page 3

Supplemental Figure 1 Page 4

Supplemental Figure 2 Page 5

Supplemental Figure 3 Page 6

Supplemental Figure 4 Page 7

Supplemental Table 1. Unadjusted and Adjusted HRs for ACM in each direction of maximum myocardial motion during first pass at stress

| Direction of cardiac motion | Unadjusted HR [95%CI] | p value |  | Adjusted HR | p value |
| --- | --- | --- | --- | --- | --- |
| Superior to inferior, mm | 0.85 [0.83-0.87] | <0.001 |  | 0.93 [0.91-0.95] | <0.001 |
| Lateral to septal, mm | 0.90 [0.87-0.93] | <0.001 |  | 0.97 [0.94-1.00] | 0.036 |
| Base to apex, mm | 0.98 [0.96-1.01] | 0.238 |  | 1.01 [0.98-1.03] | 0.555 |

ACM, all-cause mortality; CI, confidence interval; HR, hazard ratio.

Supplemental Figure 1. Correlation plots of quantitative variables


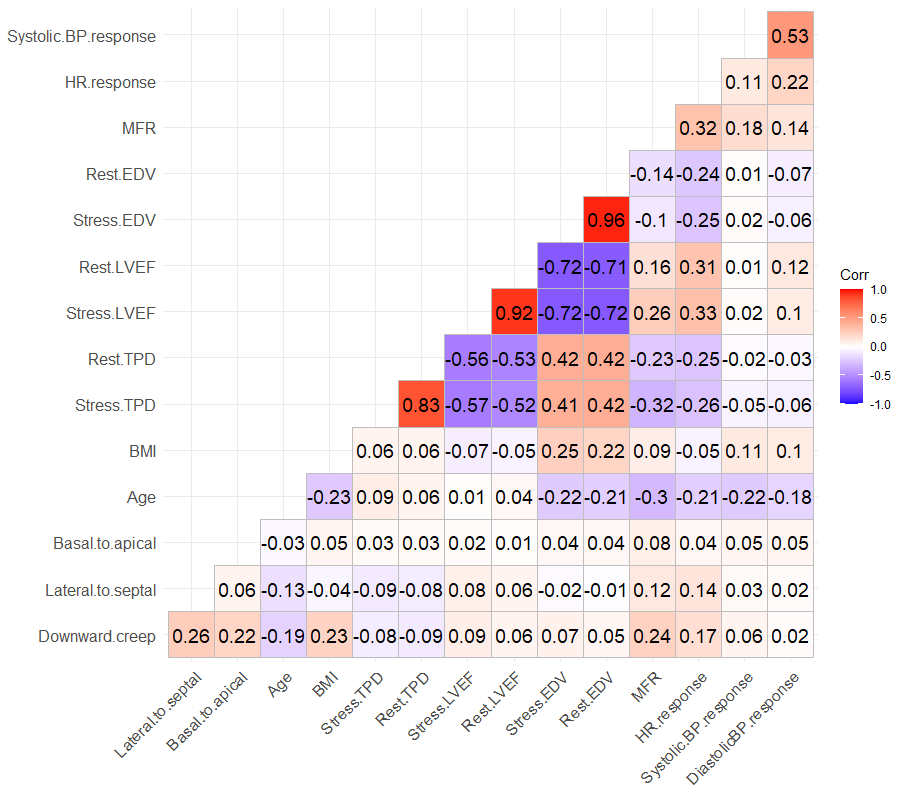


Bottom line shows correlation (Corr) coefficients between downward myocardial creep and other variables, including other directions of myocardial motion. BMI, body mass index; BP, blood pressure; EDV, end diastolic volume; HR, heart rate; LVEF, left ventricular ejection fraction; MFR, myocardial flow reserve; TPD, total perfusion deficit.

Supplemental Figure 2. Adjusted hazard ratio for all-cause mortality of downward creep modeled with restricted cubic splines


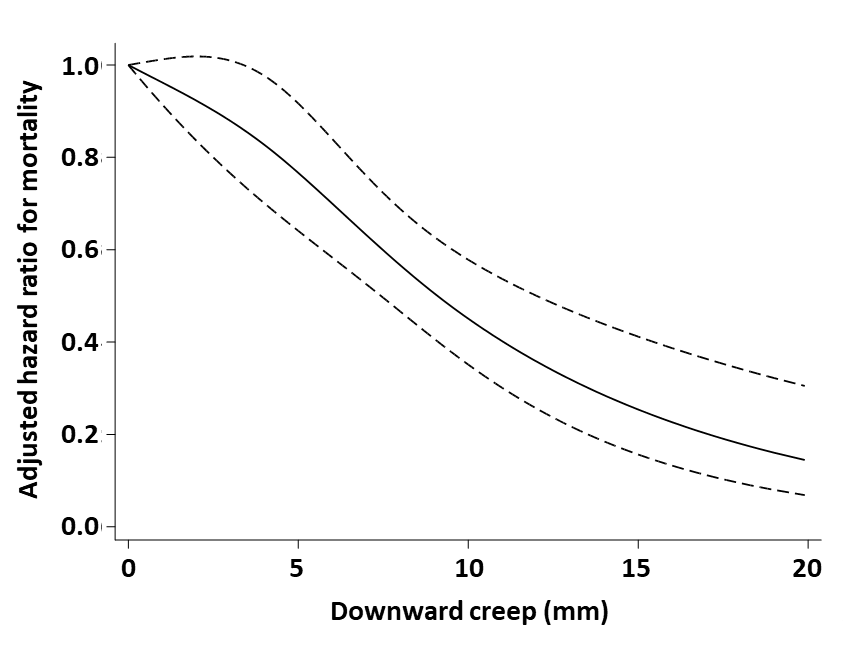


The x axis shows downward cardiac motion in mm and y axis shows hazard ratio for morality with 95% CI (dotted lines).

Supplemental Figure 3. Improvement in model fit with downward creep to predict mortality beyond conventional MPI variables and MFR


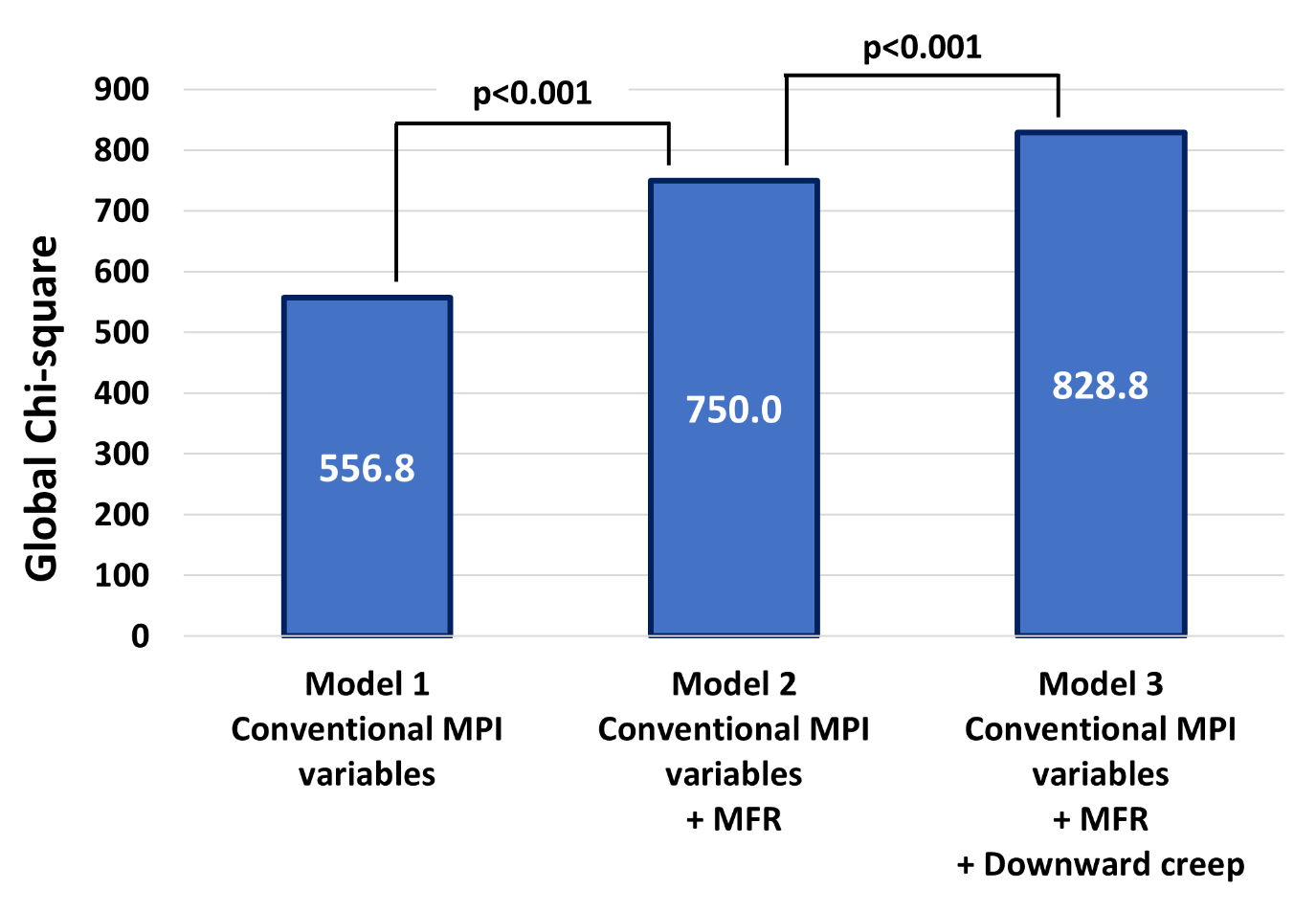


Conventional MPI variables include stress TPD, rest TPD, stress-rest change in LVEF, rest LVEF, rest LVEDV, heart rate response, systolic BP response, and diastolic BP response. BP, blood pressure; LVEDV, left ventricular end-diastolic volume; LVEF, left ventricular ejection fraction; MFR, myocardial flow reserve; MPI, myocardial perfusion image; TPD, total perfusion deficit.

Supplemental Figure 4. Improvement in model fit with downward creep to predict mortality beyond conventional MPI variables and stress MBF


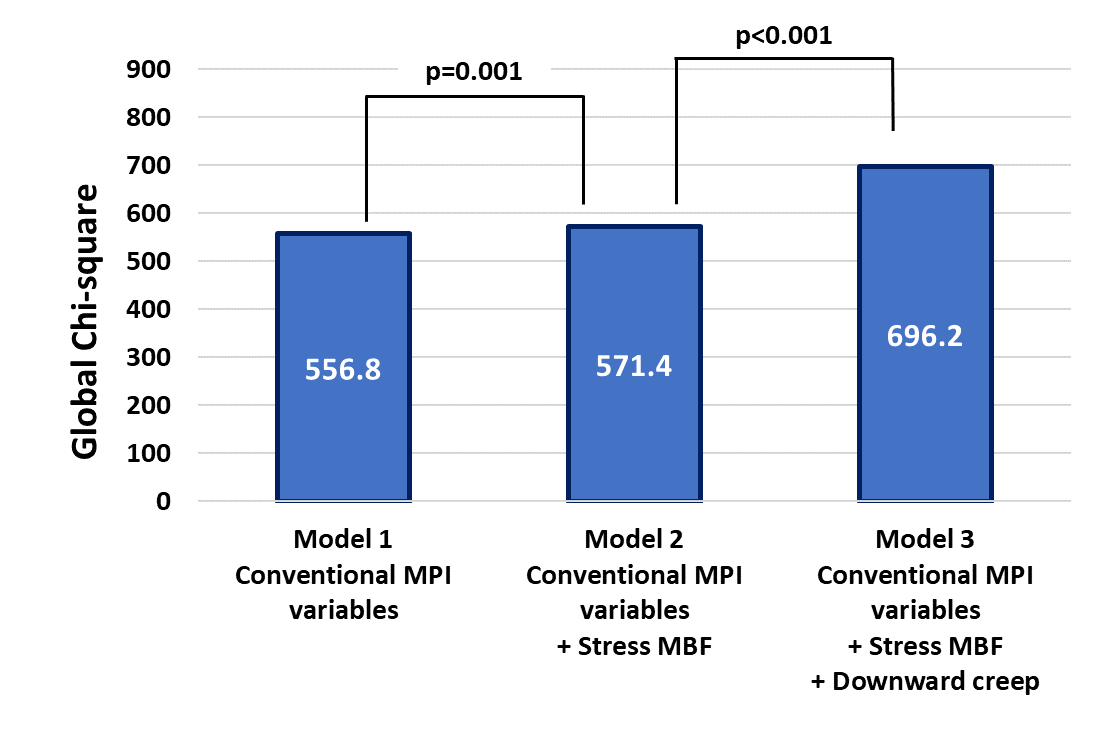


Conventional MPI variables include stress TPD, rest TPD, stress-rest change in LVEF, rest LVEF, rest LVEDV, heart rate response, systolic BP response, and diastolic BP response. BP, blood pressure; LVEDV, left ventricular end-diastolic volume; LVEF, left ventricular ejection fraction; MBF, myocardial blood flow; MPI, myocardial perfusion image; TPD, total perfusion deficit.
